# Anesthetics disrupt brain development via actions on the mTOR pathway

**DOI:** 10.1080/19420889.2018.1451719

**Published:** 2018-04-09

**Authors:** Jing Xu, Eunchai Kang, C. David Mintz

**Affiliations:** a Department of Anesthesiology, Second Affiliated Hospital of Xi'an Jiaotong University School of Medicine, Xi'an, Shaanxi, China; b Department of Anesthesiology, The Johns Hopkins University School of Medicine, Baltimore, MD, USA; c Department of Neuroscience and Mahoney Institute for Neurosciences, Perelman School for Medicine, University of Pennsylvania, Philadelphia, PA, USA

**Keywords:** mTOR, cell signaling, anesthesia, neurogenesis, neurotoxicity

## Abstract

Experiments conducted in non-human primates have recently provided new evidence supporting a longstanding concern that exposure to general anesthesia during late intrauterine life or early childhood can cause lasting cognitive deficits through harmful effects on brain development. The mammalian target of rapamycin (mTOR) signaling system plays a key role in both normal brain development and in a wide range of developmental disorders that are characterized by cognitive deficits. Intriguingly, our recently published work shows that anesthetics can chronically alter mTOR signaling in the hippocampal dentate gyrus and that normalization of mTOR signaling can prevent anesthesia-induced perturbation of structure and function. In this addendum, we briefly discuss the putative role of mTOR in developmental anesthetic neurotoxicity.

## 1. Introduction

The United States Food and Drug Administration has recently required that 12 commonly used anesthetic and sedative agents with mechanisms of action on NMDA and GABA receptors carry labels warning that repeated or lengthy exposure to these drugs between the third trimester and the first three years of life may result in adverse consequences for brain development [1]. These safety concerns are based on a combination of findings from human subjects research and animal model studies including rodents and primates. While the only prospective studies of pediatric patients conducted to date indicate that a short, single exposure to general anesthesia does not result in cognitive deficits [2,3], several large retrospective studies have found an association between anesthesia/surgery exposure in young children and worsened cognitive function at later ages [4–6]. The interpretation of the medical relevance of these studies is complex at best, and it may never be possible to design an ethical clinical study that answers fundamental questions about what kind of harm may come from anesthetic exposure. Multiple independent investigations have concluded that early postnatal exposure to general anesthesia in rodents can cause lasting deficits on behavioral tests on learning and memory e.g. [7–10]. It should be noted that some studies in rodents have found no effects of anesthetics on cognitive function [11] and others have even found neuroprotective effects [12,13]. There is considerable variability between models of early developmental anesthesia which complicates the interpretation of the rodent literature, and any roent model is potentially complicated by the need for high doses to cause general anesthesia, the difficulty in selecting an appropriate control condition, and the possibility of physiologic perturbation such as hypoxia, hypercarbia, hypotension, and hypothermia. The most compelling evidence in support of developmental anesthetic neurotoxicity comes from a recent study in non-human primates by Baxter and co-workers that showed clear deficits in visual recognition memory resulting from clinically relevant exposures to general anesthesia. These data are free from the confounds of surgery and co-morbid disease which complicate the interpretation of clinical investigations and the model for anesthesia exposure much more closely resembles human pediatric general anesthesia than the published rodent models [14]. The mechanisms by which general anesthetics might disrupt brain development have yet to be clearly defined. Early work on this topic identified molecular evidence of upregulation of pro-apoptotic pathways by general anesthetic drugs in the developing brain [15–17], but more recent studies have cast doubt on whether cell death is the principal mechanism of damage in anesthetic neurotoxicity [18–20]. Thus, it is of interest to consider other potential mechanisms of injury, particularly those which relate developmental anesthetic neurotoxicity to better-studied disorders of brain development. In this addendum to our recently published work, Kang et al 2017, we briefly discuss mTOR dysregulation in neurodevelopmental disease and we place our findings in the context of two other recent studies related to anesthetic neurotoxicity.

## 2. The mTOR pathway and neurodevelopmental disease

The mTOR protein is a highly conserved protein kinase which is the key participant in two protein complexes, mTOR complex 1 and 2 that play a critical role in growth, metabolism, and homeostasis in almost every eukaryotic cell type. mTOR complexes regulate the integrated response of multiple signaling pathways to a wide array of intracellular sensing cues and extracellular signals [21]. In the developing nervous system, mTOR plays a key role in a wide variety of normal processes, including neural stem cell regulation, cellular migration, and dendrite, axon and synapse development [22]. Thus, it is unsurprising that perturbations in mTOR signaling are potentially pathological, although given the complexity of the pathway it can be difficult to determine whether changes in mTOR signaling are the cause of pathology or a consequence of it. One of the best-studied examples of the role of aberrant mTOR activity in the pathology of neurodevelopmental disease is in tuberous sclerosis, a multisystem genetic disorder that can include intellectual disability, developmental delay, and autism spectrum disorder (ASD). Tuberous sclerosis results from mutations in TSC1 and TSC2, which are direct negative regulators of the mTORC1 complex [23,24]. Transgenic mouse models of tuberous sclerosis such as Tsc2+/− and Tsc1+/− exhibit increased activity in the mTOR pathway, which is accompanied by deficits in long-term potentiation and spatial learning, which can be reversed by chronic treatment with a rapamycin, a mTOR pathway inhibitor [25]. Another well-characterized example is Fragile X syndrome, the most common developmental cause of mental retardation and a common cause of ASD, which results from the transcriptional silencing of the FMR1 gene. Elevated mTOR signaling is observed in the Fmr1-/- mouse model of Fragile X Syndrome e.g.[26], however, the connection between Fmr1 and mTOR has not been fully elucidated. While later treatment with rapamycin does not necessarily reverse pathology associated with the Fmr1 knockout [26], a double knockout that also deletes S6 Kinase does not show the synaptic and behavioral deficits associated with Fmr1-/- alone [27]. A role for mTOR has also been postulated for a variety of neuropsychiatric disorders, including other causes of ASD, schizophrenia, drug addiction and depression [28]. While changes in the mTOR pathway in these disorders are not simple or uniform, the trend is generally that inappropriately increased activity is associated with pathology.

## 3. mTOR as a target in developmental anesthetic neurotoxicity

Because the mTOR pathway is involved in many processes brain development, any pharmacologic action on this signaling pathway has the potential to cause a pathologic state. The first report that a widely used general anesthetic acts on the mTOR pathway came from a study conducted in the human umbilical vein endothelial cell line, which found that isoflurane exposure increased levels of phospho-Akt, phospho-mTOR, and phospho-GSK3B and that effects of isoflurane on hypoxia-inducible factor 1 can be blocked with rapamycin [29]. Two manuscripts published this past year have provided evidence that increased activity in the mTOR pathway may contribute to the phenotype of developmental anesthetic neurotoxicity. Na and co-workers found that rapamycin treatment prevented the loss of chemotaxis that results from developmental exposure of C. elegans to isoflurane. Based on this data and the results of a forward genetics screen that identified endoplasmic reticulum stress pathways as a target in anesthetic-induced neurotoxicity, that isoflurane acts on mitochondria to create reactive oxygen species that in turn activate the mTOR pathway [30]. In our recently published manuscript, we described a study in a mouse model of developmental anesthetic neurotoxicity which revealed that anesthetic effects on mTOR signaling profoundly disrupted development of newborn dentate gyrus granule cells in the hippocampus. Isoflurane exposure was found to cause a lasting increase in phospho-S6 (pS6) levels that was accompanied by a reduction in mushroom spines. Both the spine loss and behavioral deficits in learning induced by anesthetic exposure could be reversed by treatment with rapamycin. We concluded that isoflurane caused a chronic upregulation of mTOR that disrupted the developmental program in this vulnerable cohort of neurons, which are critical for learning functions [10].

Of note, several previous reports suggest that anesthetics may act to suppress mTOR activity in the hippocampus in rodent models of anesthetic neurotoxicity, a finding that may or may not be at odds with ours given differences in study design and in what was measured. For example, work in both primary cultured hippocampal neurons and in the intact hippocampus, Li et al. found that isoflurane exposure during development acutely reduced phospho-S6 via inhibition of the Insulin-like growth factor 1(IGF-1)/ Phosphoinositide 3-kinase (PI3K)/ Protein Kinase B (Akt) pathway [31]. Furthermore, they found that that treatment with IGF-1 on the day of exposure and for two days afterward restored performance on behavioral tests of learning that were impaired with isoflurane exposure in their model. There are, of course, differences in the exposure paradigm between this report and ours, but perhaps the most striking difference is that we measured pS6 levels at 12 and 42 days after the exposure, rather than immediately after the exposure. Liu et al., working in an aged rodent model rather than during development, found that sevoflurane exposure resulted in a decrease in phospho-mTOR levels in the hippocampus that persisted for 7 days after exposure [32]. However, no later time points were measured, and intriguingly they found that sevoflurane exposure did not result in any change in pS6 levels between 1 and 7 days of exposure. The model in this study differs very substantially from our own in terms of the aged versus developing hippocampus, but certainly these findings do not support a universal or uniform effect of anesthetics on the mTOR pathway.

It is not year clear how to reconcile the currently published studies currently on the effects of anesthetics on the mTOR pathway. Some of the difficulty results from a broader problem in the field of developmental anesthetic neurotoxicity where there is currently no standard model of anesthetic exposure nor any agreed upon standard for measurable outcomes and time points at which these outcomes are considered relevant. Nevertheless, there is now preliminary evidence suggesting that anesthetics can have unintended and harmful effects on the mTOR pathway, and given the importance of this pathway for brain development, we feel that this new avenue of investigation deserves further consideration.
